# Susceptibility of *Spodoptera frugiperda* to Commercial Entomopathogenic Fungi Formulations in South Africa

**DOI:** 10.3390/insects16070656

**Published:** 2025-06-24

**Authors:** Simoné Louw, Vongai M. Paradza, Johnnie van den Berg, Hannalene du Plessis

**Affiliations:** IPM Program, Unit for Environmental Sciences and Management, North-West University, Potchefstroom 2531, South Africa

**Keywords:** *Beauveria bassiana*, biopesticides, fall armyworm, *Metarhizium anisopliae*, pest management

## Abstract

Chemical control using synthetic insecticides is the most widely used method for controlling the fall armyworm (FAW), *Spodoptera frugiperda* (J.E. Smith) (Lepidoptera: Noctuidae) in Sub-Saharan Africa (SSA). However, the application of insecticides is not a long-term or sustainable solution. Biological control is an important pillar of integrated pest management, and entomopathogenic fungi (EPFs) are becoming increasingly important as biocontrol agents. However, no EPF biopesticides have been registered in South Africa for the control of *S. frugiperda*. Few studies have been conducted on the efficacy of commercial formulations of biopesticides against all *S. frugiperda* life stages. The objective of this study was, therefore, to assess the potential of two *Beauveria bassiana* (Balsamo-Crivelli) Vuillemin (Hypocreales: Clavicipitaceae) and two *Metarhizium anisopliae* (Metschnikoff) Sorokin (Hypocreales: Clavicipitaceae) based commercial biopesticides registered in South Africa against other pests, for their efficacy against *S. frugiperda*. The effects of these EPF formulations were evaluated on larval and prepupal mortality, moth emergence, fecundity, and longevity of the emerged moths. The results indicated that *S. frugiperda* second- and sixth-instar larvae were not susceptible to the biopesticides. Moth emergence, fecundity, and longevity were not significantly affected. However, prepupae were susceptible to both *Metarhizium* formulations, with *Metarhizium anisopliae* ICIPE 78 resulting in the highest mortality (56.7%). This biopesticide holds potential for the management of *S. frugiperda* when applied to the soil for the control of pupating larvae.

## Simple Summary

The fall armyworm remains an important pest globally, causing substantial crop losses, particularly in maize. Sub-Saharan Africa’s favorable climate supports the proliferation of this highly adaptable pest in the region. In response, widespread and indiscriminate pesticide use has led to the emergence of resistant populations of fall armyworms. Its migratory behavior, high reproductive rate, and cryptic feeding habits further complicate chemical control, emphasizing the need for integrated pest management strategies. Biological control offers a sustainable approach that enables natural pest population regulation without harming beneficial organisms. Several laboratory studies have demonstrated the efficacy of entomopathogenic fungal isolates against different developmental stages of the fall armyworm. While numerous laboratory studies have demonstrated the effectiveness of entomopathogenic fungal (EPF) isolates against various life stages of the fall armyworm, few have evaluated the efficacy of commercially available EPF-based biopesticides. This study investigated the pathogenicity of commercial biopesticides to fall armyworm larvae and pupae. Biopesticides were ineffective against the fall armyworm larvae. However, prepupae were susceptible to biopesticides, resulting in higher pupal mortality and malformations in emerging moths. These findings highlight the potential of using commercial biopesticides as soil treatments to manage the fall armyworm during its pupation phase.

## 1. Introduction

The introduction of the fall armyworm (FAW), *Spodoptera frugiperda* (J.E. Smith) (Lepidoptera: Noctuidae), into Sub-Saharan Africa (SSA) led to significant damage to maize (*Zea mays* L.) (Poaceae) and poses a significant concern for food security [1,2]. Maize yield loss across different African countries ranged from 11% to 58% in the 2017–2019 cropping seasons, translating to USD 9.4 billion in annual losses [3,4]. Chemical control remains the most frequently utilized method for its control [5,6,7,8]; however, it is not a long-term and sustainable solution, as the adaptability of the pest enables it to continuously evolve resistance to various pesticides [9,10,11,12,13,14]. *Spodoptera frugiperda* is expected to remain a significant pest, especially in areas where resident populations occur [13]. An integrated pest management (IPM) approach provides a valuable framework for effective management [1].

Biological control using entomopathogenic fungi (EPF) is rapidly emerging as a potential alternative to synthetic insecticides for controlling *S. frugiperda* [15,16,17,18]. Entomopathogenic fungi are any member of the fungal kingdom that can infect arthropods and insects, causing their death or reduced fitness [19]. The most commonly used biopesticides are based on *Beauveria bassiana* (Balsamo-Crivelli), Vuillemin (Hypocreales: Clavicipitaceae), and *Metarhizium anisopliae* (Metschnikoff) Sorokin (Hypocreales: Clavicipitaceae) [20]. They are known to effectively produce a variety of toxin-induced infections in suitable hosts [21]. Mycotoxins, such as beauvericins and bassianolides *from B. bassiana* and dextruxins produced by *M. anisopliae*, act as immunosuppressants to facilitate host infection [22,23]. Entomopathogenic fungi have various advantages over synthetic insecticides. They occur naturally and have a low risk of infecting non-target beneficial organisms, making them environmentally safe [24,25,26]. Entomopathogenic fungi can also infect non-feeding life stages of insect pests. This is ascribed to their unique mode of infection, as EPFs can infect hosts through cuticle adhesion without requiring ingestion. It also enables them to attack various groups of arthropods, such as sap-sucking mites and ticks [27,28]. Nevertheless, EPFs also have disadvantages, including sensitivity to environmental and biological factors, such as temperature, humidity, and solar radiation, which influence their successful infection of a host [29,30,31,32,33]. The formulation of EPFs with inert materials such as fillers, dispersants, and surfactants into technical concentrates, wettable powders, and oil formulations serves to boost virulence, increase shelf life and field persistence, and allows for easier delivery of the biological control agent [31,34]. The enhanced infectivity of *B. bassiana* and *M. anisopliae* formulated in oil rather than water [35,36,37] is ascribed to the broader distribution and greater adherence of the oil to the insect cuticle, potentially enabling it to reach intersegmental membranes [29].

The development and implementation of biopesticides in SSA are still in their early stages [13,38]. Currently, no EPF biopesticides are registered in South Africa for *S. frugiperda* [39]. However, the successful use of EPFs such as *B. bassiana* and *M. anisopliae,* as seen in several other countries demonstrates the reliability of this biotechnology [15,16,17,18]. The commercialization of EPFs for use in maize production systems could provide an additional control option against *S. frugiperda* in SSA [13]. Most laboratory-based screening studies have shown high mortality rates in *S. frugiperda* larvae using aqueous EPF suspensions. There are very few studies that have been done on the efficacy of commercial formulations of bioinsecticides against all *S. frugiperda* life stages in SSA.

We hypothesized that EPF formulations would be effective against all instars and life stages of *S. frugiperda.* Therefore, this study aimed to assess the efficacy of two B. bassiana (Broadband^®^ and Eco-Bb^®^) and two M. anisopliae-based commercial biopesticides (Real Metarhizium 69^®^ and Real Metarhizium 78^®^) registered in South Africa against other crop pests, against *S. frugiperda*. The objectives of this study were to determine the efficacy of four EPF formulations for controlling *S. frugiperda* larvae and prepupae and their sublethal effects on adult emergence, fecundity, and longevity.

## 2. Materials and Methods

### 2.1. Rearing of Spodoptera frugiperda

The population used in this study was established as a reference population collected by research personnel from Corteva Seeds in 2017, soon after *S. frugiperda* invaded South Africa. Sampling was performed in a maize field at Makoppa, Thabazimbi, in the Limpopo province (24°32′44.268″ S; 27°11′5.564″ E) (designated Mk-0117). Five hundred larvae (parental generation) were reared for 80 consecutive generations before being donated to the North-West University (NWU) in Potchefstroom to serve as a reference population for future research. The population was rejuvenated in the 88th generation with 100 larvae collected in small-scale farmer’s fields in the Venda region (23°1′36.472″ S; 30°26′24.061″ E), where no insecticides had been used for their control. Larvae were reared individually in small plastic containers with aerated plastic mesh-infused lids (55 mm in length × 25 mm in diameter) due to their cannibalistic nature. Stonefly *Heliothis* premix diet (Ward’s Natural Science Establishment, LLC, Rochester, NY, USA) was prepared with distilled water added to the premix powder in a 4:1 ratio and provided to larvae as food. The containers were kept in a rearing room at 28 ± 1 °C, 60–65% RH and a 14L:10D photoperiod. The larvae were transferred to clean containers with fresh food every third day until pupation. Pupae were removed from the rearing containers and placed in a Petri dish lid (90 mm diameter) on the bottom of a clean oviposition container and aerated with a mesh-infused lid (400 mm long × 300 mm wide × 170 mm high). Male and female moths that eclosed from the pupae were mated in these containers. Three small bottles closed with a cotton plug and containing a 10% sucrose solution served as food sources for the moths. Three wax paper sheets (300 mm × 300 mm) were placed in the container for oviposition. Oviposition containers were examined daily, and the wax paper sheets were replaced. Egg batches were placed separately into transparent plastic containers, which were aerated with steel mesh-infused lids (55 mm in height × 25 mm in diameter) and kept in the same rearing room under the conditions described above. The larvae that emerged were maintained in these containers until they reached the third instar. The larvae were then transferred individually into small plastic containers (as described above) until they pupated.

### 2.2. Entomopathogenic Fungi Formulations Used in This Study

Four commercial products that are not currently registered for the control of *S. frugiperda* in South Africa were used in this study. Three of these were emulsifiable oil (ES) formulations, viz. Broadband^®^, Real *Metarhizium* 69^®^, Real *Metarhizium* 78^®^, and the other, a wettable powder (WP) formulation, Eco-Bb^®^. The complementary information on the products is listed in Table 1. The working concentrations of the biopesticides used in the bioassays were derived from their maximum recommended rates for field application, as per the product label, based on a spray volume of 300 L/hectare (ha).

### 2.3. Fungal Viability Assessment

The formulations were stored under the recommended storage conditions in a dark, cold room at 4 °C to maintain viability. Viability testing of the respective EPF formulations was conducted once at the beginning of the study, according to the methods described by [16]. Fungal suspensions were prepared by mixing 1 mL of the emulsifiable oil formulation with 9 mL sterile distilled water containing 0.05% Triton X-100 in universal glass bottles containing five 5-mm glass beads. The bottles were vortexed to produce a homogeneous conidial suspension. For the wettable powder formulation, a 3 × 10^6^ spores/mL suspension was prepared by measuring the concentration using a Neubauer-improved hemocytometer. Viability was determined by spread plating 0.1 mL of conidial suspension on Potato Dextrose Agar (PDA) for *B. bassiana* formulations and Sabouraud Dextrose Agar (SDA) for *M. anisopliae* formulations. Three replicate plates were used for each formulation. The Petri dishes were incubated at 25 ± 2 °C in the dark. Conidial germination was examined after 24 h of incubation. For each Petri dish, the percentage germination was calculated by counting the number of germinated conidia per hundred randomly selected conidia in a field covered by three coverslips under a microscope at 40× magnification. Conidia with twice the diameter of the conidia and those with visible germ tubes were scored as viable. An EPF formulation was considered viable if germination exceeded 70% [40].

### 2.4. Susceptibility of Second- and Sixth-Instar Spodoptera frugiperda Larvae to Bioinsecticides

The susceptibility of second- (L2) and sixth-instar (L6) *S. frugiperda* larvae was evaluated using a leaf dip bioassay. Maize leaves (Hybrid: DKC 80-10) were cut into 50 mm × 50 mm pieces and dipped into 200 mL of each EPF formulation at the desired working concentration (Table 1). The control leaf pieces were dipped in sterile distilled water containing 0.05% Triton X-100. The leaf pieces were air-dried. One leaf piece was then transferred to each Petri dish (90 mm in diameter), and an individual larva was transferred onto the leaf piece. The Petri dishes were incubated at 25 ± 2 °C and 65% RH under a 16L:8D photoperiod. Thirty larvae of each instar were used per replicate, and each treatment was replicated three times. The treatments were laid out in a completely randomized design (CRD). The larvae were supplied with fresh maize leaf material every second day. The leaf pieces were treated again on the fourth day, as indicated on the respective biopesticide labels. Larval mortality was recorded daily for 7 days. Insects were considered dead if they did not move when stimulated with a fine-tip brush. A mycosis test was performed on the dead larvae to confirm fungal infection. The cadavers were surface sterilized with 70% alcohol and rinsed thrice with distilled water. The cadavers were placed in Petri dishes lined with moist, sterile filter paper sealed with Parafilm. The Petri dishes were kept in an incubator at 25 ± 2 °C in the dark for 7–14 days to allow fungal growth [16]. Mortality due to fungal infection was confirmed by the presence of hyphae and conidia on the surface of the cadavers.

### 2.5. Effect of Bioinsecticides on Spodoptera frugiperda Adult Emergence

The L6 larvae that survived beyond the 7-day period were provided with food until pupation to evaluate the sublethal effect of the respective biopesticides on *S. frugiperda* adult emergence. Pupae were removed on the day of pupation and monitored for moth emergence for 12 days. Fifteen pupae (unsexed) were collected per replicate. Three replicates were performed for each treatment, arranged in a completely randomized design (CRD). Each replicate was placed in a 500 mL plastic container (150 mm in height × 150 mm in diameter) covered with a mesh net. The number of adults that eclosed in each container was recorded daily. The total number of adults that eclosed, failed to eclose, and malformed adults were recorded.

### 2.6. Susceptibility of Spodoptera frugiperda Prepupae to Bioinsecticides

Bioassays on prepupae were conducted according to the methods described by Erasmus et al. [41], with some modifications. The assays were conducted in 500 mL plastic containers (150 mm in height × 150 mm in diameter) covered with a mesh net. The soil was mixed with a commercially available seedling mix (Culterra, Johannesburg, South Africa) at a 1:1 ratio. The soil mixture was autoclaved at 121 °C for 15 min and allowed to cool and aerate. The sterile soil mixture (200 g) was added to transparent plastic containers to a depth of 8 cm, inoculated by spraying with 10 mL of a biopesticide formulation, and mixed thoroughly by hand to obtain a homogenized inoculated substrate. Distilled water containing 0.05% Triton X-100 was used as the control. Twenty 2- to 3-day-old prepupae were placed onto the substrate in each container and covered with a 2 cm layer of substrate to simulate natural pupation conditions. Three replicate containers were used for each treatment. Containers were covered with a perforated lid and maintained at 28 ± 1 °C and 65% RH with a 14 L:10D photoperiod. After four days, the soil was reinoculated with 10 mL of the biopesticide, as per the biopesticide application instructions. Mortality during the prepupal and pupal stages and adult emergence was recorded. The number of adults that eclosed per container was recorded daily for 15 days. Mycosis tests were performed on the cadavers of the prepupae and pupae, as described previously.

### 2.7. Effect of Bioinsecticides on Fecundity and Longevity of Spodoptera frugiperda

Based on the prepupal mortality results, one formulation for each fungal species, viz. Ecobb (WP) and Mt 78 (ES) were selected for this bioassay. The experimental setup, soil inoculation, and treatment arrangement were the same as described in Section 2.6. *Spodoptera frugiperda* pupae, 2- to 3-day-old, were sexed by examining the position of the genital openings under a light microscope, as described by Ref. [42]. Groups of male and female pupae were separately exposed to the treated soil. Twenty male and female pupae were used per replicate to ensure that sufficient adults eclosed for the bioassays. Pairs of female and male moths that emerged on the same day from the same treatment group were transferred to new 500 mL transparent plastic containers (150 mm in height × 150 mm in diameter) for oviposition. Eight pairs were used per replicate, and three replicates were used per treatment (*n* = 24 pairs). The containers were covered with a mesh net and maintained at 28 ± 1 °C, 65% RH with a 14 L:10D photoperiod. The moths were provided with a 10% sucrose solution on cotton balls placed in 50 mL plastic tubes inside the containers. A piece of wax paper was placed in the containers as an oviposition substrate, which was inspected for egg batches and replaced daily. Eggs that were laid on the side and mesh net of the plastic containers were carefully removed using a fine paintbrush. One-day-old eggs were counted using a light microscope. The eggs were counted daily until the females died. The longevity of each individual insect was also recorded.

### 2.8. Data Analysis

Statistical analyses were performed using the R statistical package [43]. When the control group data had only zeros (L2 and L6 mortality and mycosis data), the control group was excluded from the analyses. All binary data (conidial viability; larvae and prepupa mortality; adult emergence and mycosis) were fitted to a generalized linear model (GLM) with a binomial distribution, followed by Tukey’s HSD post-hoc test at *p* < 0.05. The mean fecundity per female was analyzed using a GLM model assuming a negative binomial distribution to account for overdispersion. Survival analysis based on the Kaplan−Meier product-limit method was used to determine the survival probability functions of moths exposed to different treatments and controls. Pairwise log-rank tests were conducted to compare the survival function curves for the different treatments. Survival data were grouped to compare the longevity of males, females, male and female pairs of each treatment group, and the individual insect groups from all treatments.

## 3. Results

### 3.1. Viability Assessment

The viability of all EPF formulations used in this study exceeded 75%. However, the viability of the four formulations differed significantly (χ^2^ = 258.1; df = 3; *p* < 0.001). The formulation with the highest viability was Bband (96.9%), compared to Ecobb, Mt 69, and Mt 78, which had similar viability scores of between 75.4–77.8%.

### 3.2. Susceptibility of Second- and Sixth-Instar Larvae to Biopesticides

The efficacy of the four EPF formulations was low against both L2 and L6 larvae (Table 2). Second-instar larval mortality did not differ among the four EPF treatments (χ^2^ = 0.66; df = 3; *p* > 0.05), with mortalities ranging from 10.0% to 13.3%. Similarly, the EPF formulations caused between 4.4% and 8.9% mortality of L6 larvae, with no significant differences between treatments (χ^2^ = 1.65; df = 3; *p* > 0.05). No mortality was observed in the control group for either larval stage. Generally, higher mycosis levels were observed in cadavers treated with *B. bassiana* formulations than with *M. anisopliae* formulations (Table 2).

### 3.3. Effect of Biopesticides on Spodoptera frugiperda Adult Emergence

The proportion of moths that emerged from L6 larvae previously fed on EPF-treated leaves did not differ significantly from the proportion that emerged from the control treatment (χ^2^ = 2.20; df = 4; *p* > 0.05). However, a significantly higher number of malformed moths emerged from larvae fed on leaves treated with the *B. bassiana* formulations (Ecobb and Bband) than from those fed on leaves treated with the *M. anisopliae* formulations (Mt 69 and Mt 78), which did not differ from the control treatment (χ^2^ = 9.83; df = 4; *p* = 0.04) (Table 3). Malformed moths were characterized by failure to eclose, partial eclosion, twisted, and partially malformed wings.

### 3.4. Susceptibility of Spodoptera frugiperda Prepupae to Bioinsecticides

*Spodoptera frugiperda* prepupae that were exposed to EPF-inoculated soil varied in susceptibility to the different EPFs (χ^2^ = 25.78; df = 4; *p* < 0.001) (Table 4). The highest mortality of prepupae occurred in the Mt 69 and Mt 78 treatments was observed. Mortality in the Mt 78 treatment was significantly higher than that in the two *Beauveria* and control treatments. However, prepupal mortality in the two *Metarhizium* treatments (Mt 78 and Mt 69) was not significantly different. Low, but similar mortalities occurred in the *Beauveria* treatments, viz. Bband (28.3%) and Ecobb (23.3%), which did not differ significantly from the percentage mortality in the control group (18.3%). However, the mycosis level did not differ significantly among the four treatments (χ^2^ = 1.41; df = 3; *p* > 0.05). The mycosed *B. bassiana* prepupae exhibited white fungal growth, indicating white muscardine disease, while mycosed *M. anisopliae* prepupae showed green fungal growth, indicating green muscardine disease.

### 3.5. Effect of Biopesticides on Fecundity and Longevity of Spodoptera frugiperda

The fecundity of *S. frugiperda* moths exposed to the different EPF treatments during the pupal stage was not affected (χ^2^ = 0.33; df = 2; *p* > 0.05). The mean fecundity of moths that emerged from Ecobb-inoculated soil was 1088.0 ± 157.3 eggs/female, Mt 78 1153.5 ± 120.9 eggs/female, and the control group was 1240.1 ± 136.4 eggs/female.

**Figure 1:**
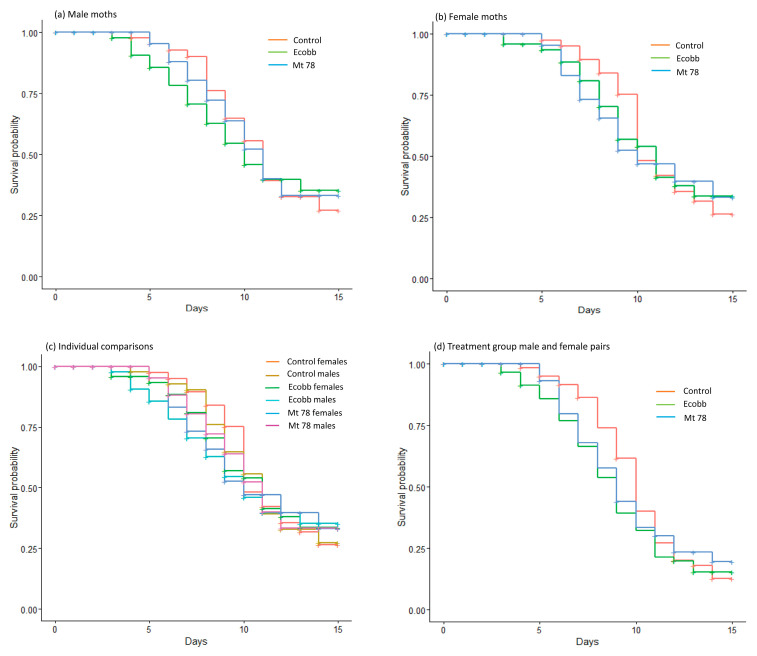
Plots of survival probability estimated for *S. frugiperda* moths after exposing the pupae to soil inoculated with *Beauveria bassiana* (Ecobb) and *Metarhizium anisopliae* (Mt 78) and for moths from the untreated control group. (**a**) Females, (**b**) males, (**c**) individual groups from all treatments, and (**d**) male and female pairs of each treatment.

Comparison of different treatment groups showed that the biopesticide formulations to which pupae were exposed did not influence the longevity of the *S. frugiperda* moths; female moths (log-rank: χ^2^ = 0.07; df = 2; *p* > 0.05) (Figure 1a), male moths (log-rank: χ^2^ = 0.17; df = 2; *p* > 0.05) (Figure 1b), individual groups from all the treatments (log-rank: χ^2^ = 0.26; df = 5; *p* > 0.05) (Figure 1c), and male and female pairs of each treatment (χ^2^ = 1.39; df = 2; *p* = 0.05) (Figure 1d).

## 4. Discussion

This study evaluated the efficacy of commercial formulations of EPFs against various developmental stages of *S. frugiperda.* Second- and sixth-instar *S. frugiperda* larvae were not susceptible to the four commercial EPF formulations used in this study (Bband, Ecobb, Mt 69, and Mt 78). These findings are comparable to the results of similar studies [44,45,46], which recorded mortality ranges of 10–14.9% by *M. anisopliae* and *B. bassiana* isolates against L2 *S. frugiperda* larvae, respectively. Similarly, low mortality levels of 3.3–5.6% were also recorded for L6 larvae by Idrees et al. [44]. The methods used for EPF application and fungal concentrations were similar and within the ranges used in the current study, allowing for an accurate comparison between the results. However, several studies have reported high efficacy levels of EPFs against *S. frugiperda* larvae, pupae, and adults [47,48,49,50,51]. This could possibly be ascribed to differences in bioassay methodology used in the latter studies, for example, differences in fungal concentrations, method of fungal delivery, strain pathogenicity, and fungal formulation. In addition, inter- and intra-species variations in pathogenicity/virulence among EPFs are linked to differences in enzymatic and molecular characteristics [51]. Entomopathogenic virulence genes involved in different infection processes, such as host adherence, cuticle degradation, colonization, and host immune system suppression, are genetically regulated [52]. Differences in the expression and specificity of some virulence factors influence genetic variability [53,54]. Maistrou et al. [55] found that the more virulent strains of *B. bassiana* produced higher levels of oospores in a mycotoxin with antimicrobial and cytotoxic effects—and the catalase enzyme (Cat P), which enhances the fungus’s ability to adapt to oxidative stress. Overexpression of cell wall-degrading enzymes, such as chitinases and proteases, leads to increased infection efficiency [52]. In some instances, some strains may lack virulence factors against specific target insects [54]. Although the biopesticides used in this study are not registered against *S. frugiperda* in South Africa, the rationale for their evaluation was based on them being registered against other lepidopteran pests such as the African bollworm ((*Helicoverpa armigera* (Hübner) (Lepidoptera: Noctuidae)), false codling moth (*Thaumatotibia leucotreta* (Meyrick) (Lepidoptera: Tortricidae)) and potato tuber moth (*Phthorimaea operculella* (Zeller) (Lepidoptera: Gelechiidae)).

Notable differences were observed in mycosis levels resulting from the different treatments. Despite similar mortality levels, *B. bassiana* formulations exhibited greater sporulation than those of *M. anisopliae.* Higher mycosis levels were observed in L2 and L6 larvae fed on leaves treated with B. bassiana formulations than in those fed on leaves treated with *M. anisopliae* formulations. Mycosis is an important fitness characteristic of EPF because sporulation allows for the self-propagation of the fungus in the environment [56]. In addition to direct infection, EPFs are known to cause a significant reduction in feeding in some insects, resulting in insect death. This is attributed to EPF toxins that may deter insects from feeding further [57]. Alternatively, toxins can prevent further feeding by disrupting the insect’s structural integrity and physiological processes [58]. Reduced feeding has been demonstrated in larvae of the cotton leafworm, *Spodoptera littoralis* (Boisduval) (Lepidoptera: Noctuidae) [58] and *S. frugiperda* [44]. This may explain the observed low mycosis rates with some treatments despite similar mortality levels. However, feeding performance assessments were not conducted in this study, which could have allowed the death of non-mycosed individuals to be attributed to non-feeding.

When *S. frugiperda* prepupae were exposed to inoculated soil, there was a higher mortality rate with *M. anisopliae* formulations. The mycosis levels were similar, indicating successful infection in all four treatments. Soil is a known habitat for EPFs, including *Metarhizium* and *Beauveria* species [59]. As such, EPFs may perform better in soil rather than foliar spray applications since soil acts as a natural reservoir providing nutrition that allows for EPF proliferation [60]. The potential of soil drenching as an application method for EPFs to control the soil-dwelling stages of *S. frugiperda* also seems to be a more practical field application method, as the soil protects the fungus from ultraviolet radiation [18]. However, since sterile soil was used in this experiment, field evaluations are necessary to validate the results because the persistence of EPFs depends on several other factors, such as soil physical properties, ambient temperature, moisture, and the presence of other competing microorganisms [61].

The evaluation of emergence, malformation, fecundity, and adult longevity to assess the sublethal effects of EPFs on *S. frugiperda* fitness yielded interesting results. While there was no difference in adult emergence in the control treatment, prepupae exposed to *B. bassiana* showed significantly higher malformation, similar to the results of Montecalvo et al. [33]. It has been reported that EPFs can produce toxins that can result in improper cuticle formation, leading to malformations [62]. *Metarhizium anisopliae* and *B. bassiana* produce toxins that cause the opening of Ca^2+^ channels in the membrane, which disrupts chitin synthesis, leading to deformities [63]. Abnormalities in adults, such as reduced body and wing size and low flight ability, result in reduced mating, leading to a low reproductive potential of the population [64,65]. However, exposure of *S. frugiperda* pupae to EPFs did not cause any significant sublethal effects in the adults that emerged, possibly due to the sclerotic pupal shell, which acts as a barrier against fungal infection [64,66].

## 5. Conclusions

*Spodoptera frugiperda* larvae were not susceptible to the commercial biopesticide formulations used in this study, and adult emergence, fecundity, and longevity were not significantly affected. However, prepupae were susceptible to *Metarhizium* and *Beauveria* formulations, leading to higher pupal mortality and physiological costs in the form of malformations. Despite the low mortality, potentially greater pest control is possible by exploiting the sublethal effects of EPFs. Further research on *M. anisopliae* ICIPE 78, which is already commercialized in South Africa, is needed to validate its efficacy against *S. frugiperda* prepupae in the soil. It may have the potential for the management of *S. frugiperda* when applied as a soil treatment to control pupating larvae. Further trials are needed to determine the efficacy of the selected formulations in natural field soils compared with the sterile soils used in this study. Assessing their environmental performance and persistence under varying abiotic and agronomic conditions will provide valuable insights into their adaptability and potential for integration into pest management strategies that target *S. frugiperda*.

## Figures and Tables

**Table 1:** Commercial entomopathogenic fungal formulations screened against larvae and pupae of *Spodoptera frugiperda*.

Commercial Name and Manufacturer	Active Ingredient	Experimental Code	Application Rate/ha	Batch Concentration	Working Concentration
Broadband^®^, BASF SA (Pty) Ltd.^©^) (Midrand, South Africa)	*Beauveria bassiana* PPRI 5339	Bband	1000 mL	4 × 10^9^ cfu/mL	1.33 × 10^7^ * cfu/mL
Eco-Bb^®^, Andermatt PHP (Pty) Ltd.^©^) (uMngeni, Nottingham Road, South Africa)	*Beauveria bassiana* R444	Ecobb	1000 g	2 × 10^9^ spores/g	3.5 × 10^6^ spores/mL
Real *Metarhizium* 69^®^, Real IPM SA (Pty) Ltd.^©^) (Grabouw, South Africa)	*Metarhizium* *anisopliae* ICIPE 69	Mt 69	400 mL	1 × 10^9^ cfu/mL	1.33 × 10^6^ cfu/mL
Real *Metarhizium* 78^®^, Real IPM SA (Pty) Ltd.^©^) (Grabouw, South Africa)	*Metarhizium* *anisopliae* ICIPE 78	Mt 78	400 mL	1 × 10^9^ cfu/mL	1.33 × 10^6^ cfu/mL

* cfu = colony-forming units.

**Table 2:** Percentage mortality and mycosis of second- and sixth-instar *Spodoptera frugiperda* larvae treated with entomopathogenic fungal formulations.

	L2 Larvae		L6 Larvae	
Treatment	% Mortality (±SE)	% Mycosis (±SE)	% Mortality (±SE)	% Mycosis (±SE)
Bband	11.1 ± 4.84 ^a^*	100 ± 0.0 ^d^	4.44 ± 1.11 ^a^	33.3 ± 33.3 ^a^
Ecobb	10.0 ± 5.09 ^a^	72.2 ± 14.7 ^c^	8.89 ± 1.11 ^a^	77.8 ± 11.1 ^ab^
Mt 69	10.0 ± 1.92 ^a^	36.1 ± 7.35 ^a^	5.55 ± 2.22 ^a^	0 ± 0.0 ^a^
Mt 78	13.3 ± 3.33 ^a^	22.2 ± 11.1 ^ab^	5.55 ± 4.01 ^a^	25.0 ± 25.0 ^a^
χ^2^	0.66	8.64	1.65	10.1
*p*	>0.05	<0.001	>0.05	=0.01

* Means within columns followed by the same letter do not differ significantly at *p* < 0.05 (Tukey’s HSD post-hoc test).

**Table 3:** Percentage emergence and malformed *Spodoptera frugiperda* moths from sixth-instar larvae treated with entomopathogenic fungal formulations.

Treatment	% Emergence (±SE)	% Malformation (±SE)
Control	80.0 ± 3.85 ^a^*	8.37 ± 0.40 ^ab^
Bband	66.7 ± 6.67 ^a^	21.3 ± 7.23 ^c^
Ecobb	75.6 ± 11.1 ^a^	20.2 ± 8.94 ^c^
Mt 69	75.6 ± 8.01 ^a^	2.78 ± 2.78 ^a^
Mt 78	75.6 ± 2.22 ^a^	3.03 ± 3.03 ^a^
χ^2^	2.20	9.83
*p*	>0.05	=0.04

* Means within a column followed by the same letter do not differ significantly at *p* < 0.05 (Tukey’s HSD post-hoc test).

**Table 4:** Percentage mortality and mycosis of *Spodoptera frugiperda* prepupae exposed to soil inoculated with different entomopathogenic fungi (EPF) formulations.

Treatment	Mean Mortality ± SE (%)	Mean Mycosis ± SE (%)
Control	18.3 ± 6.01 ^a^*	–
Bband	28.3 ± 4.41 ^ab^	33.3 ± 33.3 ^a^
Ecobb	23.3 ± 4.41 ^ab^	23.3 ± 14.5 ^a^
Mt 69	41.7 ± 6.01 ^bc^	27.8 ± 20.0 ^a^
Mt 78	56.7 ± 6.01 ^c^	38.3 ± 10.5 ^a^
χ^2^	25.78	1.41
*p*	<0.001	>0.05

* Means within columns followed by the same letter are not significantly different at *p* < 0.05 (Tukey’s HSD post-hoc test).

## Data Availability

The raw data supporting the conclusions of this article will be made available by the authors upon request.

## Abbreviations

The following abbreviations are used in this manuscript:

ES	Emulsifiable oil
EPF	Entomopathogenic fungi
FAW	Fall armyworm
SSA	Sub-Saharan Africa
WP	Wettable powder
